# Defining an Orbitofrontal Compass: Functional and Anatomical Heterogeneity Across Anterior–Posterior and Medial–Lateral Axes

**DOI:** 10.1037/bne0000442

**Published:** 2021-04

**Authors:** Ines V. Barreiros, Hironori Ishii, Mark E. Walton, Marios C. Panayi

**Affiliations:** 1 Department of Experimental Psychology, University of Oxford; 2 Nuffield Department of Clinical Neurosciences, University of Oxford; 3 Wellcome Centre for Integrative Neuroimaging, University of Oxford

**Keywords:** anterior–posterior, medial–lateral, orbital prefrontal, orbitofrontal cortex, agranular insula

## Abstract

The orbitofrontal cortex (OFC) plays a critical role in the flexible control of behaviors and has been the focus of increasing research interest. However, there have been a number of controversies around the exact theoretical role of the OFC. One potential source of these issues is the comparison of evidence from different studies, particularly across species, which focus on different specific sub-regions within the OFC. Furthermore, there is emerging evidence that there may be functional diversity across the OFC which may account for these theoretical differences. Therefore, in this review we consider evidence supporting functional heterogeneity within the OFC and how it relates to underlying anatomical heterogeneity. We highlight the importance of anatomical and functional distinctions within the traditionally defined OFC subregions across the medial–lateral axis, which are often not differentiated for practical and historical reasons. We then consider emerging evidence of even finer-grained distinctions within these defined subregions along the anterior–posterior axis. These fine-grained anatomical considerations reveal a pattern of dissociable, but often complementary functions within the OFC.

As interest and the number of publications on the function of the orbitofrontal cortex (OFC) increase (Izquierdo, 2017, Figure 1; Murray et al., 2007), inconsistences are often found, especially when comparing data from different species. A number of reviews comparing cross-species findings have emphasized that not all areas of the OFC have been equally studied (Izquierdo, 2017; Wallis, 2012) and the most targeted areas in each species are not necessarily homologous (Heilbronner et al., 2016; Rudebeck & Murray, 2011; Wallis, 2012). For example, rodent studies focus on lateral OFC, which often extends to include adjacent anterior insular (AI) cortex, both of which are agranular, whereas studies in primates typically focus on area 13, an anterior and agranular sector of central OFC. Aligning the findings from different studies is made more complicated as studies often do not explicitly specify which subregion within the OFC was the focus of the investigation (Izquierdo, 2017; Wallis, 2012).

Increasingly researchers are considering how the functional diversity within the OFC aligns with its anatomical subdivisions. This has mostly focused on established anatomical subdivisions along the medial–lateral axis (Bradfield & Hart, 2020; Izquierdo, 2017; Kringelbach & Rolls, 2004; Rudebeck & Murray, 2014; Walton et al., 2015). More recent data suggest that there may also be important functional distinctions along the anterior–posterior axis (Bradfield et al., 2018; Panayi & Killcross, 2018). Indeed, sensitivity to these anatomical distinctions will likely be critical to progress in understanding the functional role of the OFC. These anatomical considerations raise the question of where, within the OFC, should functional boundaries be drawn? And are there distinct functional divisions or should we consider these differences functional gradients instead?

One lens through which to view such questions is by focusing on anatomical studies (Devlin & Poldrack, 2007). The function of a brain region is, to a great extent, determined by the pattern of neuroanatomical connections it makes with other areas; the more dissimilar the connectivity pattern of two brain regions, the easier it should be to differentiate their functions (Passingham et al., 2002). Therefore, here, we review the evidence for functional heterogeneity first along the medial–lateral and then the anterior–posterior axes of OFC organization, describing how functional differences relate to the patterns of anatomical inputs and outputs. We will primarily focus on what has been found in rodent OFC, and selectively relate this to evidence of similar functional and organizational principles in the human and non-human primate OFC.

## Classical Parcellation of Rat OFC

**Figure 1 fig1:**
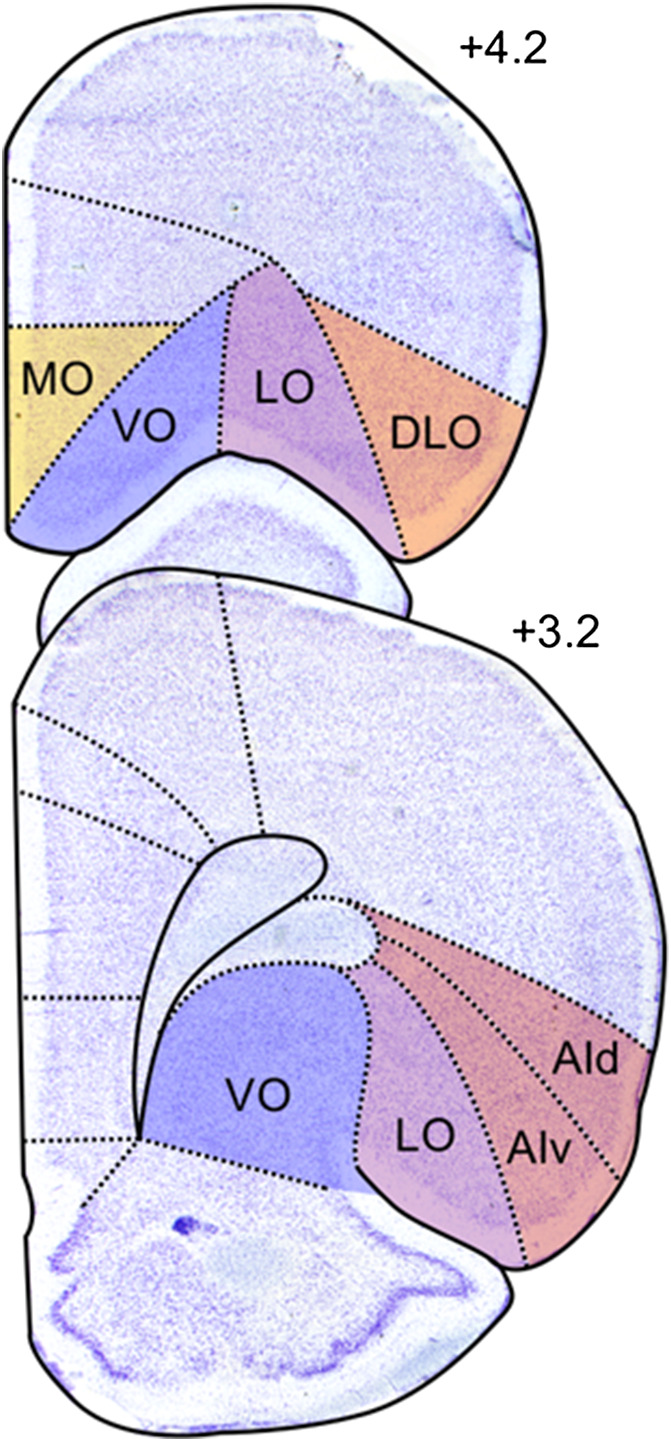
Classical Parcellation of Rat OFC *Note*. Currently recognized OFC subdivisions include MO, VO, LO, DLO, and AI. Represented is the right hemisphere of Nissl stained coronal rat brain slices at two different anterior–posterior levels of the OFC. Numbers on the right-hand side of the slices indicate the distance in millimeters from bregma, consistent with Paxinos and Watson (1998). An intermediate region, VLO (not shown) has also been identified, located halfway between VO and LO. In this focused review VLO will not be considered, and DLO will not be differentiated from AI. Photomicrographs are adapted from Panayi & Killcross (2018). AId: agranular insular cortex, dorsal part; AIv: agranular insular cortex, ventral part; DLO: dorsolateral OFC; LO: lateral OFC; MO: medial OFC; VO: ventral OFC; VLO: ventrolateral OFC. See the online article for the color version of this figure.

While the structural organization of the primate OFC has been extensively investigated (Carmichael & Price, 1994; Öngür et al., 2003; Price, 2007), the precise boundaries of rodent OFC are still under debate. The rodent OFC is situated in the dorsal bank of the rhinal sulcus and, in contrast to both the human and non-human primate OFC, which can be cytoarchitectonically defined using granular, dysgranular, and agranular areas (Price, 2006), all its regions are agranular (Van De Werd & Uylings, 2008; Wise, 2008). While borders of its various portions are indistinct cytoarchitectonically, several subdivisions of the rodent OFC are currently recognized (Figure 1). Originally, Krettek & Price (1977b) subdivided the rat orbital region into medial (MO), ventral (VO), ventrolateral (VLO) and lateral (LO) OFC, and the agranular insular cortex, lateral to LO, into dorsal and ventral sectors (AIv, AId). In their original description, the delimitation of agranular insula extended rostrally, lateral to the orbital areas. However, the AId portion rostral to the claustrum was later reclassified as dorsolateral orbital area (DLO) due to its distinct thalamocortical projections and cytoarchitecture (Price, 2006; Ray & Price, 1992). Based on structural and connectional similarities, it has been proposed that MO, VLO, LO, and DLO may be comparable to areas 14, 13a, 13 m/L, and 12o, respectively in monkeys, while AIv and AId may correspond to monkey insula areas Iam/Iapm and Iai (Price, 2006).[Fig fig1]

In practice, which regions are included under the umbrella of OFC proper have not been agreed upon in rodents or primates. For example, in rodents, AI is often targeted alongside adjacent LO (e.g. Ogawa et al., 2013), and has been considered an orbital structure, whereas MO is not always considered part of rodent OFC (e.g. Schoenbaum et al., 2006). Similarly, in primates, there is debate over the status of parts of area 12 (Murray & Rudebeck, 2018; Price, 2007; Sallet et al., 2020). Here we will not attempt to reach any conclusions as to which regions should be considered part of the OFC, and instead will inclusively consider the functional and anatomical distinctions of all potential OFC subregions along the orbital surface in rodents, which for simplicity we will refer to as MO, VO, LO, and AI.

## Medial Versus Lateral Distinctions

A series of influential neuroanatomical studies in primates gave rise to the idea that the anatomical organization of medial and orbital parts of prefrontal cortex can be aggregated into two functional networks with partially overlapping but distinct connectivity patterns (revised in Ongur & Price, 2000; Price, 2007). Specifically, there is a “medial visceromotor” network comprising predominantly of parts of the medial OFC (area 14) and the medial frontal cortex (as well as some caudolateral parts of OFC), and an “orbital sensory” network largely comprising the central and lateral OFC in primates (centered on areas 11, 13, and 12). While these divisions are primarily based on studies of non-human primates (NHPs), based on putative cytoarchitectonic and connectional similarities between rodent OFC and caudal parts of primate OFC, a similar division has also been proposed to be present in rodents (Ongur & Price, 2000; Price, 2007).

It is therefore not surprising that there has been a focus on examining whether there are functional correlates of this medial–lateral distinction in primates and, more recently, in rodents. In rodents, there is now mounting causal evidence that MO and LO play distinct roles in a range of functions including cost–benefit decision making (St. Onge & Floresco, 2010; Stopper et al., 2014), reversal learning (probabilistic spatial reversal learning, Dalton et al., 2016; Verharen et al., 2020; delay-discounting with spatial reversal, Mar et al., 2011; serial visual reversal learning, Hervig et al., 2020), and drug reinstatement (Arinze & Moorman, 2020; Fuchs et al., 2004). While there are many differences between the studies, it appears that MO disruption particularly affects choices when values have to be integrated and compared, whereas LO disruption has a bigger effect on value updating in similar circumstances. In addition, it is notable that LO disruption only impairs behavioral flexibility in Pavlovian stimulus-outcome learning (Gallagher et al., 1999; Gardner et al., 2017; Panayi & Killcross, 2018; Schoenbaum et al., 2003), whereas MO disruption instead impairs behavioral flexibility in instrumental action-outcome learning (Bradfield et al., 2015; Gardner et al., 2017, 2018; Gourley et al., 2016; Ostlund & Balleine, 2007; Panayi & Killcross, 2018). This pattern of dissociable medial–lateral functions is also supported by electrophysiological recordings of neuronal activity in LO and MO showing distinct representations of task parameters (e.g. size of predicted reward in a delay discounting task, Burton et al., 2014; Roesch et al., 2006; trial type, epoch within a trial and value state representation in a Pavlovian unblocking task, Lopatina et al., 2017), with neurons in MO tracking value representation independent of context and LO tracking contextual features independent of value.

Functional dissociations between medial and lateral OFC subdivisions have also been observed in both NHP and human studies (e.g. Kringelbach & Rolls, 2004). Again, the most consistent differences have been observed in tasks where subjects make cost–benefit decisions or need to exhibit behavioral flexibility (Rudebeck & Murray, 2011; Walton et al., 2011). While the precise functions of these networks are beyond the scope of this review, there appear encouraging commonalities with rodent studies. For instance, medial OFC is more commonly associated with value integration and comparison and with internally guided choices, whereas lateral OFC appears more closely linked with sensory processing and updating specific stimulus–reward associations (Howard et al., 2015; Noonan et al., 2010, 2017; Rudebeck & Murray, 2011; Walton et al., 2010).

There is also evidence for an anatomical underpinning to the medial–lateral distinction in rodents, which follows the connectivity patterns observed in primates. For example, Heilbronner et al. (2016) point out that rodent MO, like primate medial OFC, is well placed to form part of a medial motivational/visceromotor network based on projections to medial and ventral striatum while VO/LO, like NHP central/lateral OFC, projects to the central striatum and thus could comprise a sensorimotor network. Similarly, just as in NHPs, there is a gradient of distinct mediodorsal thalamic inputs in rodents that suggest separate visceromotor and sensory networks from medial to lateral OFC respectively (Krettek & Price, 1977a; Price, 2006; Ray & Price, 1993; Reep et al., 1996; Shi & Cassell, 1998). Moreover, VO and LO, but not MO, are reciprocally connected to primary and secondary somatosensory areas (Reep et al., 1996), and both, along with AI, receive projections from piriform cortex, suggesting a strong association between these lateral regions and a sensory network (Datiche & Cattarelli, 1996).

However, there are a number of findings that raise questions about how straightforward this homology is. For example, while perirhinal cortex connections are primarily restricted to the more central/lateral orbital network in NHPs, all OFC subregions in the rat share reciprocal projections with the perirhinal cortex (Agster & Burwell, 2009; Kondo et al., 2005; Price, 2006). Likewise, projections to the periaqueductal gray and lateral hypothalamus in the rat are not only from MO and VO, corresponding to similar projections from a medial visceromotor network in NHPs, but also from AI (Floyd et al., 2000, 2001; Hoover & Vertes, 2011; Öngür et al., 1998). Furthermore, the exact boundary of a medial and lateral network in rodents is under debate. For example, while VO is often included in the lateral network (Heilbronner et al., 2016), Hoover & Vertes (2011) instead suggest that rodent VO should be linked more closely with MO as part of a medial network given that they share similar projection patterns and both regions have only weak projections to LO.

Therefore, while rodent MO and LO appear to map neatly onto a medial–lateral connectional distinction, adjacent VO and AI regions do not. Note, however, that even in NHPs some medial and lateral OFC regions are classified as belonging to both the medial and orbital networks (Carmichael & Price, 1995; Kondo et al., 2005; Price, 2007). Notably, several of these “intermediate interface” areas in primates are agranular, which have been proposed to be the most likely homologs with rodent agranular OFC regions (Wise, 2008). Therefore, while further systematic tracing studies are needed to map out rodent OFC networks, the extant evidence suggests that broadly similar medial and lateral orbital networks might exist in rodents and NHPs (Rudebeck & Murray, 2011; Wallis, 2012).

## Beyond a Dichotomous Mediolateral Distinction?

Despite the compelling overlap between anatomical and functional evidence for separate medial and lateral orbital networks, it remains an open question whether this is the most appropriate level of granularity to consider OFC functions. For example, as described above, while a lateral orbital network might include VO, LO, and AI, there is increasing evidence that these VO and AI regions may have distinct and even dissociable functions compared to LO.

Even though LO and AI are putatively classified as part of the lateral orbital connectivity network, there is evidence that they have different functions. For example, Ishii and colleagues showed that LO and AI have bidirectional effects on risky decision-making: LO inactivation increases risk preference in rats during a gambling task, whereas AI inactivation decreases it (Ishii et al., 2012). In a subsequent study, they showed that these effects are mimicked by 5-HT1a receptor antagonists in LO or AI (Ishii et al., 2015). Given the interconnections between these areas, Ishii and colleagues speculate that the areas may interact to ensure an appropriate balance of behavioral policies after gains or losses for AI and LO, respectively. Moreover, chemogenetic-induced inhibition of posterior AI impaired the expression of goal-directed behavior, whereas VO/LO inhibition only disrupted goal-directed behavior when there was a change in the identity of the outcome (Parkes et al., 2018). This suggests that the recruitment of VO/LO in action-outcome learning depends on specific task structure, whereas AI plays a more general role in supporting goal-directed behavior.

Unfortunately, to date, few studies have directly compared the functions of VO and LO. However, lesions of rat VO but not LO were found to impair performance in a spatial reference memory task (Corwin et al., 1994) and cause attentional deficits (King et al., 1989), suggesting that there may also be a functional difference distinction between VO and LO (see Balleine et al., 2011).

Recently in NHPs, in addition to distinguishing between medial and lateral OFC (Rudebeck & Murray, 2011; Walton et al., 2010, 2011), a finer-grained distinction is emerging within lateral OFC networks. Several studies have specifically implicated the more central areas 13/11 as necessary for processing of current reward value and identity but not for behavioral flexibility (e.g. Burke et al., 2014; Rudebeck et al., 2017), whereas parts of more lateral areas, such as orbital area 12 and adjacent anterior insula are involved in credit assignment of reward value to cues and actions and implementing flexible behavioral strategies (e.g. Chau et al., 2015; Rudebeck et al., 2017; Sallet et al., 2020; Wittmann et al., 2020).

**Figure 2 fig2:**
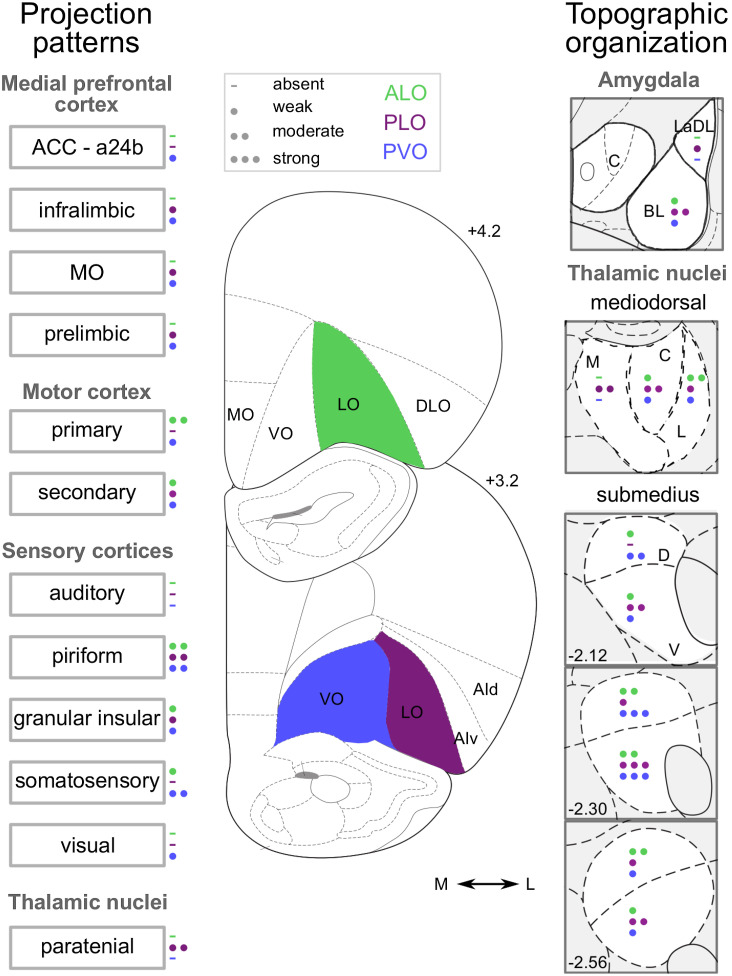
Density of Inputs Into ALO, PLO, and PVO *Note*. The different number of circles represent the average density of retrogradely labeled cells following CTB injection (i.e., absent, weak, moderate, or strong) into ALO, PLO, and PVO. Panels depicting labeling in amygdala and in MD thalamus represent the average density labeling in all slices quantified and not at that particular coronal section. Numbers on the right-hand side of the slices indicate the distance in millimeters from bregma, consistent with Paxinos and Watson (1998). Figure adapted from Barreiros et al. (2021). a24b: anterior cingulate cortex, area 24b; ACC: anterior cingulate cortex; AId: agranular insular cortex, dorsal part; AIv: agranular insular cortex, ventral part; ALO: anterior lateral OFC; DLO: dorsolateral OFC; LaDL: lateral amygdala, dorsolateral part; LO: lateral OFC; MO: medial OFC; PL: prelimbic cortex; PLO: posterior lateral OFC; PVO: posterior ventral OFC; VO: ventral OFC; BL: basolateral; C: central; M: medial; L: lateral; D: dorsal; V: ventral. See the online article for the color version of this figure.

Such functional differences appear underpinned by distinct differences in inputs to these orbital regions, as well as distinct cortico-striatal projections organized topographically along the medial–lateral axis. In rodents, for instance, in support of the dissociations observed by Ishii and colleagues (Ishii et al., 2012, 2015), AI and MO send strong projections to the nucleus accumbens, whereas LO and VO project more densely to the central striatum (Berendse et al., 1992; Heilbronner et al., 2016; Schilman et al., 2008). Additionally, AI and MO but not LO or VO receive strong dopaminergic inputs (Descarries et al., 1987). Moreover, these distinctions extend beyond a separation of AI from other lateral OFC sectors. For instance, both inputs from prelimbic and infralimbic cortices (Vertes, 2004) and outputs to the basolateral amygdala (BLA) (Gabbott et al., 2005) are strong for MO, VO, and AI, but only weak for LO. There is also a medial–lateral distinction in inputs from sensory cortices to the posterior portions of VO and LO, with VO but not LO receiving projections from somatosensory and visual areas, and both receive distinct, topographically organized projections from the mediodorsal and submedius thalamic nuclei (Barreiros et al., 2021; Reep et al., 1996; see Figure 2).[Fig fig2]

Similarly, in primates, there are also a number of anatomical distinctions between central (area 11/13) and more lateral (area 12) regions that might support functional differences. For example, in primates polymodal inputs from the superior temporal gyrus more strongly target lateral than central OFC regions (Carmichael & Price, 1995; Price, 2007), whereas perirhinal cortex projects predominantly to central OFC (Ongur & Price, 2000). Moreover, there are gradations of strength of inputs from amygdala across the mediolateral axis, with the strongest projections targeting medial and lateral regions but not central OFC.

Taken together, these studies suggest that rather than a simple lateral versus medial functional distinction, it matters where exactly in the mediolateral axis OFC function is investigated (Izquierdo, 2017; Murphy & Deutch, 2018).

## Anterior Versus Posterior Distinctions

Another important functional distinction, although so far less recognized in the field, lies along the anterior–posterior axis (see also Murray & Rudebeck, 2018). For instance, Panayi and Killcross (2018) found that, while both anterior (ALO) and posterior LO (PLO) lesions, defined anterior and posterior to the genu of the corpus collosum, impaired Pavlovian outcome devaluation, only PLO but not ALO lesions disrupted Pavlovian reversal learning. This suggests that within the anterior–posterior axis of the LO there is a dissociation of rapid flexible behavioral control and long-term updating of outcome value. Bradfield et al. (2018) have also revealed a similar anterior–posterior functional dissociation within rodent MO. They found that only lesions of the anterior but not posterior portion of MO disrupt instrumental devaluation, selectively implicating this anterior region in the control of instrumental actions based on inferring information about future unobservable outcomes (Bradfield et al., 2018).

While an anterior–posterior functional organization has also been reported in primates, this has tended to highlight a gradation in the complexity of reinforcers, moving from more primary representations in evolutionarily older posterior regions to more abstract secondary reinforcers in more anterior regions in both medial and lateral OFC (Klein-Flügge et al., 2013; Kringelbach & Rolls, 2004; McNamee et al., 2013; Sescousse et al., 2010, 2013; Smith et al., 2010). Of more relevance, and paralleling the observations by Panayi & Killcross (2018), Murray et al. (2015) found a double dissociation after selectively inactivating either anterior (area 11) or posterior (area 13) OFC in NHPs at different points of an outcome devaluation task (Murray et al., 2015). They concluded that posterior OFC appears to be required for updating outcome valuations during the selective satiety devaluation procedure, whereas anterior OFC is necessary for translating this knowledge into action for flexible behavioral control.

Given the relative lack of cytoarchitectonic distinctions in the rodent (see Van De Werd & Uylings, 2008), there is increasing interest in whether there are anatomical differences that underpin these functional distinctions. We have recently explored the possibility of anatomical distinctions between anterior and posterior LO that might underlie the functional differences observed by Panayi & Killcross (2018) by characterizing the projections to ALO and PLO and comparing them to an adjacent posterior VO (PVO) region (Figure 2, Barreiros et al., 2021). This revealed a number of distinct connectivity patterns between the regions, with the differences between ALO and PLO being as great as the distinction on the mediolateral axis between PLO and PVO. For example, the BLA, which is also implicated in processes that support outcome-guided behavior through associative learning of sensory-specific representations and predictive-cues (for reviews see Balleine & Killcross, 2006; Sharpe & Schoenbaum, 2016), sends stronger projections to PLO than to ALO (Barreiros et al., 2021). Interestingly, a similar distinction appears present in primates, with the BLA sending stronger projections to posterior than to anterior OFC (Freese & Amaral, 2009; Price & Drevets, 2010). PLO also receives stronger projections from medial prefrontal structures, including MO, prelimbic, and infralimbic cortices, than ALO (Figure 2). By contrast, ALO receives denser afferents from somatosensory and motor cortices. We also observed distinct and topographically organized patterns of thalamic inputs from the mediodorsal and submedius nuclei of the thalamus to ALO and PLO (Barreiros et al., 2021).

Similarly, Bradfield et al. (2018) showed that their observed pattern of impairments was supported by anatomical differences, with anterior MO being more strongly connected to the nucleus accumbens (NAc) core while posterior MO has a higher density of projections to the BLA (Bradfield et al., 2018). Taken together with previous studies on the effects of lesions to these target regions on flexible behavior in instrumental devaluation (Corbit et al., 2001; Shiflett & Balleine, 2010), this also suggests the existence of dissociable functional circuits from anterior and posterior MO. Anterior–posterior distinctions such as the ones we have highlighted here are likely to be present within other subdivisions of the OFC. For instance, the anterior and posterior portions of VO receive different patterns of thalamic inputs from the submedius nucleus (Barreiros et al., 2021; Kuramoto et al., 2017; Reep et al., 1996) as well as noradrenergic inputs from the locus coeruleus (Cerpa et al., 2019). Taken together, this suggests that separate anterior–posterior functional distinctions might be present throughout rodent OFC. Such functional differences might also exist between DLO and AI, which are anatomically separate along the anterior–posterior axis. Future studies selectively targeting DLO, for which there is currently a paucity of both functional and connectivity data, might help test this hypothesis.

## Conclusion

In this review, we highlight the importance of functional and anatomical differences within OFC subregions. Recent theoretical reviews have attempted to unify the significant functional differences between medial and lateral OFC regions (e.g. Bradfield & Hart, 2020; Wilson et al., 2014). However, it is increasingly clear that within this medial–lateral dichotomy there are complementary but distinct functional roles for all the classically defined orbital sub-regions. It is therefore also important to understand how the complementary functions of these subregions contribute to the overall function of the OFC. Indeed, here we also highlight emerging evidence which points to further distinctions along the anterior–posterior axis within these classically defined OFC subregions.

While sensitivity to these anatomical distinctions is increasing in the field, currently there are few studies available that explicitly target multiple subregions to enable direct comparisons of anatomical connectivity, neuronal activity, or dissociable function. This issue is not unique to OFC: for example, there is also evidence for anterior–posterior distinctions within prelimbic and infralimbic cortex (e.g. Reynolds & Zahm, 2005), which may also underlie functional differences. A recent review by Laubach and colleagues (Laubach et al., 2018) examining similar issues when comparing studies of medial frontal structures called for increased use of precise anatomical terms (once they are agreed upon) along with reporting of estimated distances from agreed landmarks such as Bregma; we would suggest that a similar rigor could also benefit OFC research to facilitate clarity and reproducibility.

While not the main focus of this review, it is also apparent that these anatomical considerations are relevant across species, and may aid in clarifying a number of outstanding questions in the literature regarding homology between rodent and primate OFC (Ongur & Price, 2000; Preuss, 1995; Rudebeck & Murray, 2011; Wise, 2008). A fine-grained anatomical consideration of functional differences between OFC subregions has also recently helped refine questions of whether the OFC is indeed necessary for reversal learning, normally considered a hallmark deficit following OFC lesions, in NHPs (Burke et al., 2014; Rudebeck et al., 2013; Sallet et al., 2020). For example, Rudebeck and colleagues (Rudebeck et al., 2013; Rudebeck & Murray, 2014) have shown that, unlike aspiration lesions, fiber-sparing excitotoxic OFC lesions in macaques do not cause reversal deficits. In contrast, excitotoxic lesions of OFC in rodents and marmosets impair reversal learning. Recently, Wittmann et al. (2020) have implicated macaque agranular insular regions in reversal learning. They propose that the discrepancies between reversal deficits in macaque, marmoset, and rodent studies may be resolved by considering the macaque agranular insular, posterior to OFC, as homologous to marmoset and rodent OFC. Indeed in rodents, deficits in adaptive responding, including reversal learning, have often been caused by targeting more posterior regions of LO and adjacent AI regions (Groman et al., 2019; Lichtenberg et al., 2017; Panayi & Killcross, 2018). An approach sensitive to these anatomical considerations could also help resolve questions about whether the rodent OFC encodes neuroeconomic value (Gardner et al., 2018, 2019; Levy & Glimcher, 2012; Padoa-Schioppa & Assad, 2008). Taken together, the evidence we review here indicates that as OFC subregions are targeted with increasing specificity both across the medial–lateral and anterior–posterior axis, many surprising functional dissociations are likely to emerge within the OFC.
